# Blepharospasm and Bradyphrenia With Infarction of the Artery of Percheron: A Case Report

**DOI:** 10.7759/cureus.31814

**Published:** 2022-11-23

**Authors:** Srikanth Adidam Venkata, Otakhon Matchanov, Sneha Adidam, Javed Jagroo

**Affiliations:** 1 Internal Medicine, Brookdale University Hospital Medical Center, New York, USA; 2 Neurology, Lenox Hill Hospital, New York, USA; 3 Internal Medicine, Howard University Hospital, Washington, DC, USA; 4 Emergency Medicine, Eric Williams Medical Sciences Complex, Champs Fleurs, TTO

**Keywords:** stroke, artery of percheron infarct, bradyphrenia, bilateral thalamic infarct, blepharospasm

## Abstract

The artery of Percheron (AOP) is a variant of the posterior cerebral circulation where a single branch of either posterior cerebral artery supplies both paramedian territories of the thalami. A stroke of the AOP has become a neurodiagnostic conundrum due to its relative rarity and vague symptoms, and, hence, a missed opportunity for recanalization treatment. The classical presentation of AOP stroke is the triad of altered mental status, vertical gaze palsy, and memory impairment.

Here, we describe a retrospective case review of a 59-year-old male presenting with confusion and slurred speech with subsequent symptoms such as blepharospasm and bradyphrenia. The initial computed tomography of the head failed to recognize the bilateral thalamic infarct which was established on day three on brain magnetic resonance imaging. Because the patient was out of the therapeutic window for thrombolysis, dual antiplatelet therapy was started. The patient made a rapid recovery to near-baseline function and was discharged to rehab services.

This case is unique with the clinical presentation of both blepharospasm and bradyphrenia being rarely found in the literature. The shared insult to the basal ganglia-thalamocortical circuits may have caused both symptoms. Physician awareness of these subtle findings can increase awareness, earlier diagnosis, and treatment of bilateral thalamic lesions and AOP strokes.

## Introduction

Artery of Percheron (AOP) has a prevalence ranging from 7% to 11%. Among the general population, AOP infarct accounts for 0.1-2% of all ischemic strokes and 4-18% of all thalamic strokes. Patients most commonly present with a triad of altered mental status, vertical gaze palsy, and memory impairment. Bilateral thalamic acute infarcts are often not diagnosed in a timely fashion as they are rarely seen on initial computed tomography (CT) of the head or are incidentally found on magnetic resonance imaging (MRI) of the brain [1]. Bilateral thalamic infarcts pose a great diagnostic challenge due to the variability in presentation and occurrence of rare symptoms which lead to a delay in clinical diagnosis and intervention. In addition, due to the bilateral nature and symmetry of its presentation on imaging, bilateral thalamic infarcts are often attributed to metabolic, degenerative, and other systemic illnesses, leading to underdiagnosed strokes [2]. Blepharospasm, defined as the spasm of the eyelids, has traditionally been linked to basal ganglia dysfunction [3]. However, there are a few reported cases of bilateral thalamic infarction associated with blepharospasm [4]. Bradyphrenia is the pathological slowing of mental processes and is more broadly used for cognitive slowing secondary to cortical and basal ganglia pathology [5]. This case describes an unusual presentation of bilateral thalamic infarction due to AOP involvement that presented with both blepharospasm and bradyphrenia. We aim to highlight blepharospasm and bradyphrenia as potential symptoms of bilateral thalamic infarcts to increase physician awareness and investigate the potential cause of these symptoms. This will lead to quicker diagnosis of AOP strokes and better outcomes.

## Case presentation

A 59-year-old male with hypertension and hyperlipidemia, non-compliant with medication, was brought to the emergency department with confusion, forgetfulness, slurring of speech for 14 hours, and one episode of urinary incontinence. The initial vitals were blood pressure (BP) 130/84 mmHg, pulse 58 beats per minute, temperature 36.6°C, respiratory rate 18 breaths per minute, SpO_2_ 99%, and body mass index 27.12 kg/m². He answered questions after prolonged pauses with a generalized paucity of facial expressions. Blepharospasm was present, and his visual acuity was 20/40 bilaterally. The remainder of his neurologic examination was unremarkable. He denied alcohol use, recreational substance abuse, any recent illnesses, and recent travel out of state or out of the country.

**Figure 1:**
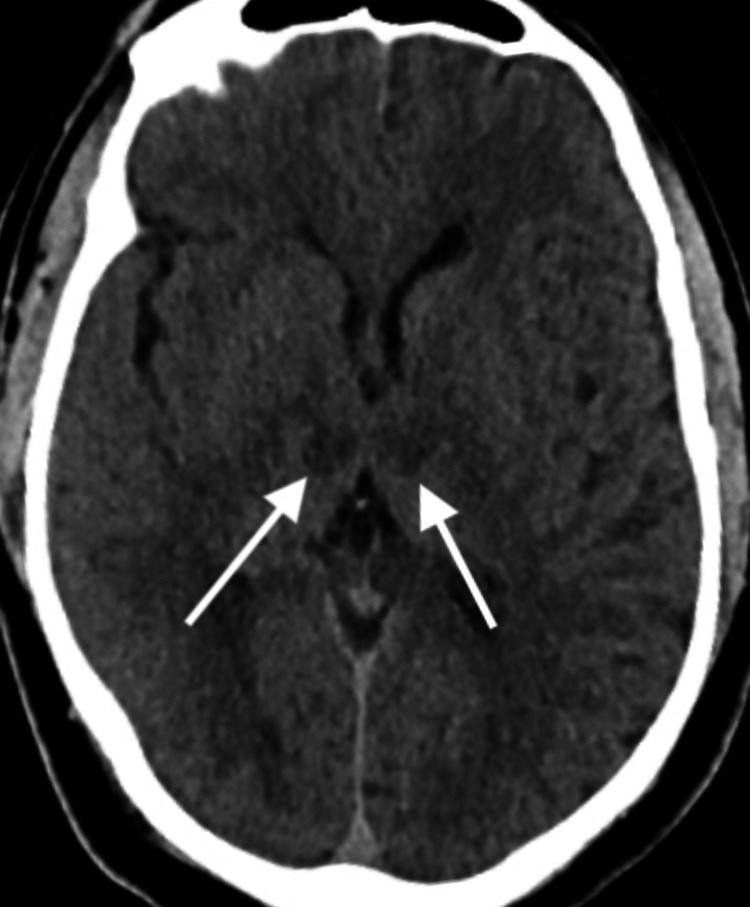
Computed tomography of the head, axial view, showing bilateral symmetric thalamic hypodensity.

A stroke code was called for acute change in mentation. He was evaluated as having a National Institutes of Health Stroke Scale score of two, and a stroke workup ensued. CT of the head was performed (Figure 1). Although initially interpreted as normal, on retrospective review, bilateral thalamic hypodensities were seen.

**Figure 2:**
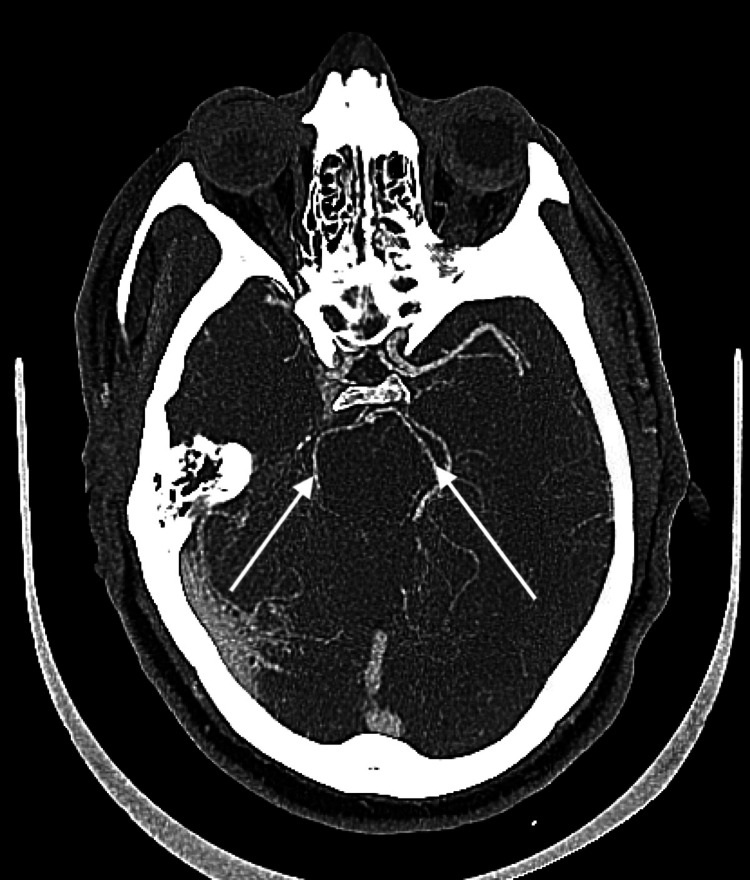
Computed tomography angiogram of the head, axial view: bilateral posterior cerebral arteries appear patent.

**Figure 3:**
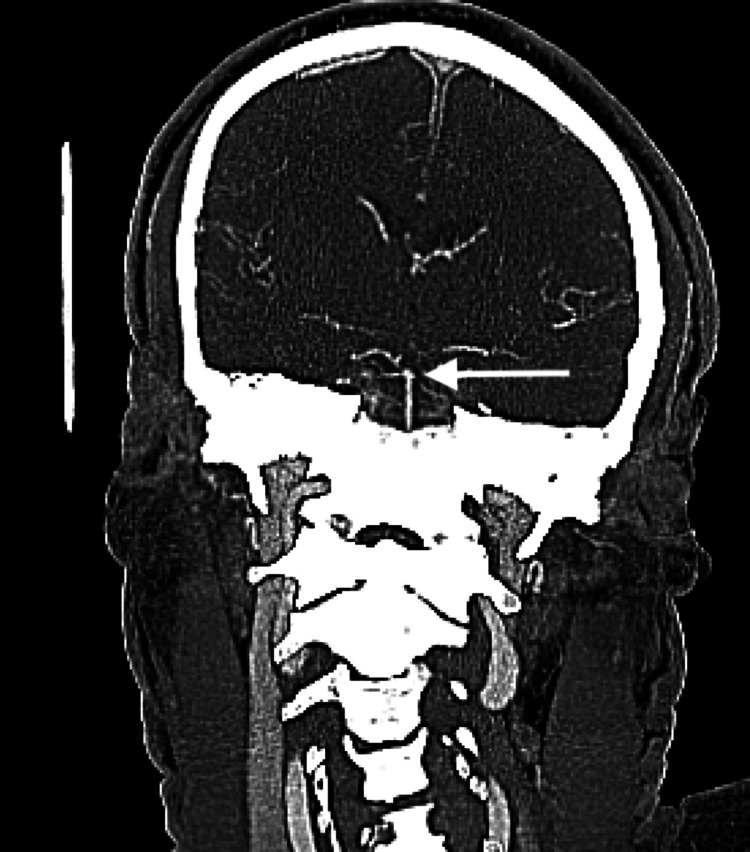
Computed tomography angiogram of the head, coronal view: top of the basilar artery appears patent.

CT angiography of the head and neck, axial view (Figure 2) and coronal view (Figure 3), demonstrated patent basilar and bilateral posterior cerebral arteries (PCAs). The patient did not receive tissue plasminogen activator (tPa) because his last known well time was 14 hours prior, and he was admitted for further evaluation of possible stroke and possible seizure.

**Figure 4:**
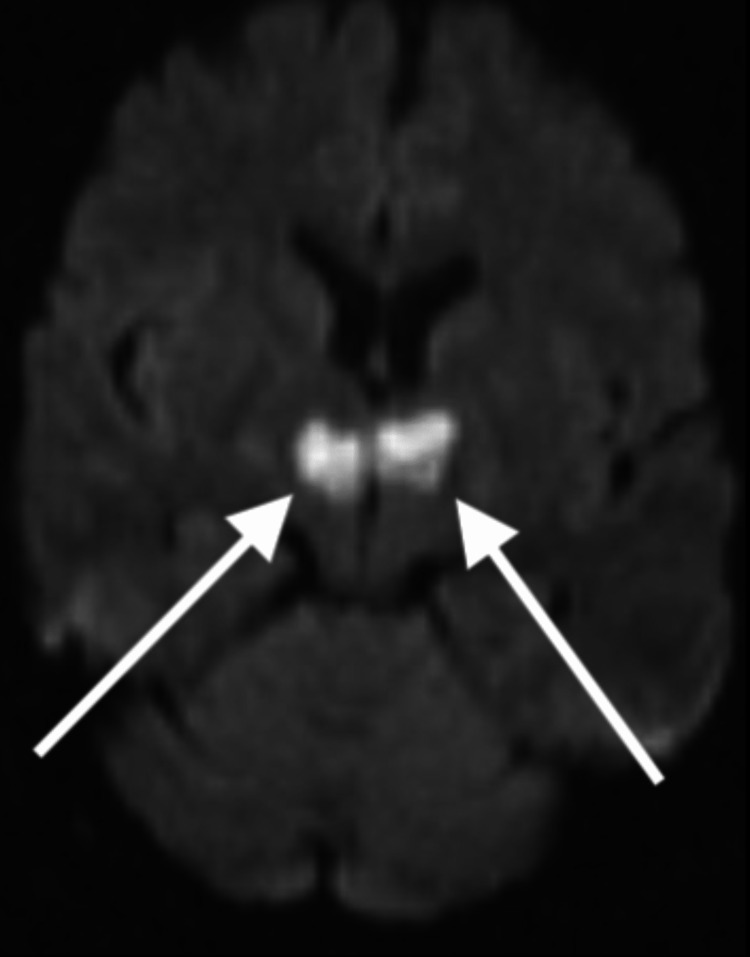
Magnetic resonance imaging of the brain, axial view: diffusion-weighted imaging showing restricted diffusion in both thalami, suggestive of infarcts.

**Figure 5:**
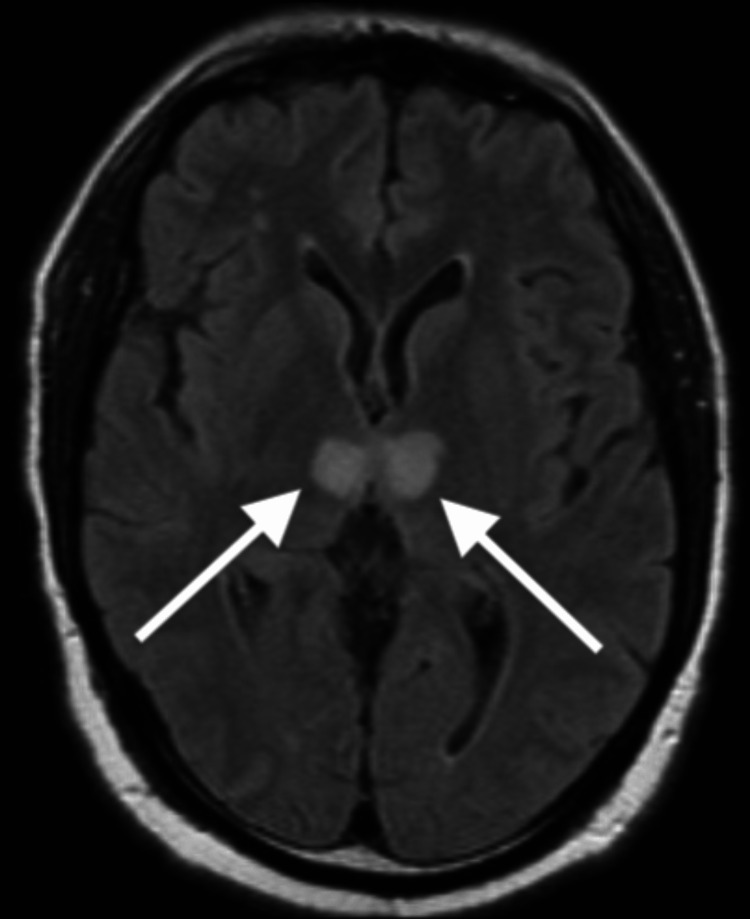
Magnetic resonance imaging of the brain, axial view: T2 fluid-attenuated inversion recovery axial view showing hypoattenuation in both thalami, suggestive of infarcts.

Levetiracetam 2 g load and 1 g every 12 hours, dual antiplatelet therapy, acetylsalicylic acid 81 mg once daily orally, and clopidogrel 75 mg for one month were recommended. Transthoracic echocardiogram, electroencephalogram, routine blood testing, and urinalysis, including toxicology screen, were within normal limits. On day three, MRI of the head diffusion-weighted imaging (Figure 4) and T2 fluid-attenuated inversion recovery (Figure 5) without contrast showed acute bilateral thalamic infarcts.

The acute bilateral thalamic infarcts were attributed to an AOP stroke. The patient made a rapid recovery to near-baseline functionality within four days with the only persisting symptom being bradyphrenia, which showed notable improvement by day seven prior to discharge.

## Discussion

Thalamic infarcts were first described by Dejerine and Roussy in 1906 [6]. The first report citing bilateral thalamic infarctions was by Beauvois and Lhermitte in 1975 [7]. In 1973, Percheron described the thalamic paramedian artery, which is now known as the AOP, a solitary branch originating from the PCA which supplies all the thalamic paramedian territories bilaterally. Studies have indicated that AOP occlusion accounts for 0.1-2% of all ischemic strokes and 4-18% of all thalamic strokes [8].

The definition of blepharospasm is eyelid heaviness; tension around the eyes; chronic, persistent eyelid spasms; and frequent blinking, which may lead to total closure of eyelids and obstruction of vision. Blepharospasm is considered a dystonic movement disorder and is commonly attributed to basal ganglia disorders but may be caused by thalamic lesions [9].

The pathophysiology of blepharospasm in thalamic lesions is poorly understood. Thalamic lesions can injure the pallidothalamic fibers in transit to the ventrolateral and ventro-anterior nuclei [10], which may produce dystonia [11]. Khooshnoodi et al. reported lesions in different regions of the brain, including the thalamus, leading to blepharospasm [12].

Bilateral thalamic injury in areas such as the paramedian territories cause decreased arousal and impaired learning and memory which may contribute to bradyphrenia [13]. Bilateral thalamic infarcts in this patient may have caused both a dysfunction in engagement in the initiation of behavior and the interruption of the complex cascade of basal ganglia-thalamocortical circuits leading to bradyphrenia [14,15].

The combination of blepharospasm and bradyphrenia in this patient, although rarely found in the literature, may be explained by the shared insult to the basal ganglia-thalamocortical circuits.

## Conclusions

High suspicion should be maintained with acute changes in cognition and new onset of subtle findings such as blepharospasm and bradyphrenia as symptoms of a bilateral thalamic lesion. Physician awareness of these symptoms can lead to earlier diagnosis of bilateral thalamic lesions and diagnosis of AOP strokes, earlier initiation of treatment, and better recovery for patients.
